# Endoscopic therapy of anastomotic diverticulum combined with stercorolith incarceration: A case report

**DOI:** 10.3389/fmed.2022.1053487

**Published:** 2022-11-29

**Authors:** Yi Liu, Zhihao Chen, Lizhou Dou, Siyao Liu, Yueming Zhang, Yong Liu, Guiqi Wang

**Affiliations:** ^1^Department of Endoscopy, National Cancer Center/National Clinical Research Center for Cancer/Cancer Hospital, Chinese Academy of Medical Sciences and Peking Union Medical College, Beijing, China; ^2^Department of Gastrointestinal Surgery, Department of General Surgery, Guangdong Provincial People’s Hospital, Guangdong Academy of Medical Sciences, Guangzhou, China

**Keywords:** endoscopic therapy, anastomotic diverticulum, diverticulosis, stercorolith, incarceration

## Abstract

Diverticulosis is a commonly acquired disease of the lower gastrointestinal tract, which may be associated with significant morbidity and adverse effects on quality of life. Although several national guidelines focused on the treatment of diverticulosis, multiple controversies remained regarding the disease management of diverticulosis. For some controversial issues, such as the role of antibiotics in mild diverticulitis, when and how to operate on patients with acute diverticulitis, there is no conclusion yet. To our knowledge, this is the first report of endoscopic therapy for anastomotic diverticulitis caused by stercorolith incarceration. In the current case, a 49-year-old woman complained of recurrent subumbilical pain without obvious inducement for half a year. Colonoscopy showed anastomotic diverticulum combined with stercorolith incarceration. After local inflammation relieved by conservative treatment, the patients received endoscopic mucosal incision and lithotomy. Then the diverticulum was closed with titanium clips. The abdominal pain of patient was completely relieved, and the reexamination of colonoscopy showed that the wound healed well after 1 year of follow-up. This case suggests that for anastomotic diverticulitis caused by stercorolith incarceration, endoscopic therapy can remove the stimulation factors better and avoid the recurrence and progression of the disease compared with conservative treatment. Moreover, endoscopic therapy achieves the maximum in minimally invasive surgery and reduces complications and surgical costs compared with radical surgery.

## Introduction

Diverticulosis is a prevalent gastrointestinal disorder that is associated with significant morbidity and health care costs in the western countries. Pathologically, colonic diverticulosis is characterized by the presence of diverticula, cystic protrusions of the mucosa and submucosa that protrude through the muscularis layer of the bowel wall at the sites of penetration of the vasa recta (1). The multifactorial pathogenesis of diverticulosis is still not fully understood. Previous studies showed that several variables that influence development include the structure and motility of the colonic wall, dietary fiber intake, genetic, and host immune responses (2, 3). In addition, several studies suggested dysbiosis, or disruption of the microbial balance in the body, may also be a potential causative factor (4, 5). There are several national guidelines on the management of diverticulosis (6–10). However, multiple controversies remain regarding the disease management of diverticulosis. In previous reported cases, sigmoid diverticula are the most common, although diverticula can occur anywhere in the colon (1, 5, 11). Anastomotic diverticulum and especially diverticulitis caused by anastomotic diverticulum combined with stercorolith incarceration were rarely seen. To our knowledge, this is the first case of endoscopic therapy for anastomotic diverticulitis caused by stercorolith incarceration, which could help clinicians develop therapeutic strategies for diverticulitis.

### Case presentation

**FIGURE 1:**
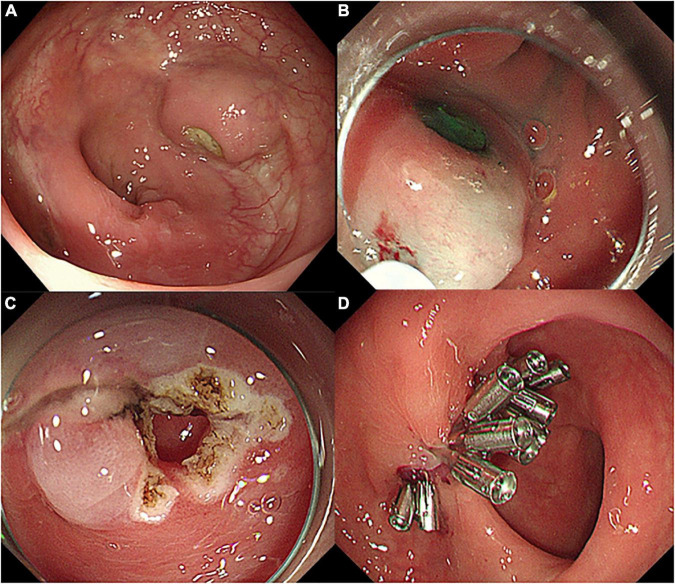
Endoscopic images of diverticulum and procedures of endoscopic therapy. **(A)** Anastomotic diverticulum combined with stercorolith incarceration, **(B,C)** endoscopic mucosal incision and lithotomy, and **(D)** diverticulum closed with titanium clips.

**FIGURE 2:**
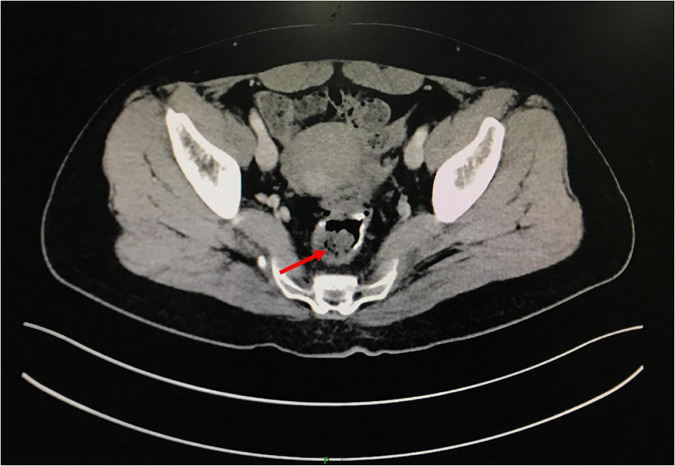
The pelvic cavity CT image.

**FIGURE 3:**
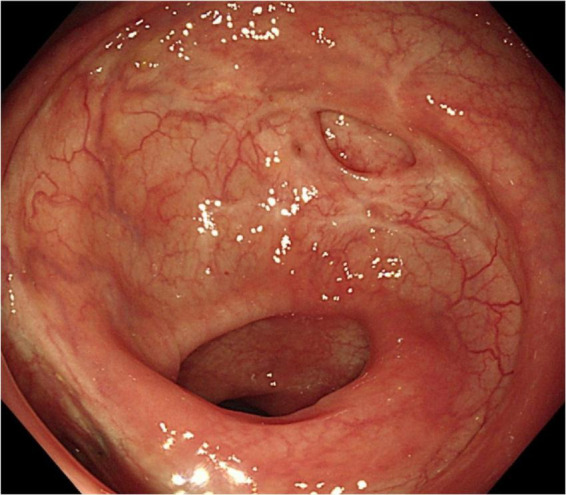
Endoscopic image of the scar after 1 year.

A 49-year-old woman complained of recurrent subumbilical pain without obvious inducement for half a year, and no discomfort such as bloody stool, diarrhea or constipation. The patient underwent radical resection for rectal cancer 14 years ago. The results of physical examination showed mild tenderness in the subumbilical abdomen without rebound pain. There were no significant abnormalities in routine blood parameters and related inflammatory indicators. Colonoscopy showed anastomotic diverticulum combined with stercorolith incarceration (Figure 1A). CT image of the pelvic cavity showed a mass of low density adjacent to the anastomosis (Figure 2). When the local inflammation was relieved by conservative treatment, the patient underwent endoscopic mucosal incision and lithotomy (Figures 1B,C). And the diverticulum was closed with titanium clips (Figure 1D). After 1 year of follow-up, the patient said that the abdominal pain was completely relieved, and the reexamination of colonoscopy showed that the wound healed well (Figure 3).

## Discussion

Diverticulitis is a prevalent gastrointestinal disorder, which may be associated with significant morbidity and adverse impact on quality of life. In most cases, patients with diverticula are asymptomatic. Diverticulitis occurs when the diverticulum becomes inflamed or infected. Symptoms include abdominal pain, fever, nausea, vomiting, cramps, and constipation (12). The pathogenesis of diverticulosis is still controversial (2, 3). As we know, colonic diverticulum is an acquired protrusion of the colonic wall. Therefore, the etiology of colonic diverticulosis is mainly related to the dysfunction of connective tissue (1). In this case, the patient underwent radical resection for rectal cancer 14 years ago. Thus, changes in colon wall structure, such as muscle thickening, enhanced collagen cross-linking, or shortening of intestinal segments, may contribute to “stiffness” predisposing to mucosal herniation. To our knowledge, no cases of anastomotic diverticulitis caused by stercorolith incarceration have been reported in the literatures before. Therefore, we pondered how to treat this patient.

Treatment of diverticulitis is determined by a variety of factors, such as disease severity, symptom burden, the patient’s general health, and complications. In general, mild cases of diverticulitis get better with dietary modifications, such as a liquid diet. If symptoms are severe or there are complications, fasting, antibiotics, or even surgery may be needed (12, 13). Destek et al. considered that surgical treatment should generally be used in patients unresponsive to conservative treatment, and in the treatment of complicated and recurrent cases (14). Other researchers found that up to 15% of patients who undergo surgical removal have relapses and up to 25% have persistent chronic pain with no signs of inflammation on imaging (15). Therefore, when and how surgery is performed in patients with diverticulitis is still uncertain. Due to recurrent episodes of diverticulitis and chronic gastrointestinal symptoms, the patient’s quality of life is severely affected. Therefore, to avoid recurring episodes of diverticulitis, closing the diverticula as soon as possible after the inflammation is under control may be ideal.

In the current case, this woman had recurrent subumbilical pain for half a year. After repeated anti-inflammatory treatment with antibiotics in other hospitals, the symptoms of abdominal pain were still repeated. When we performed colonoscopy, we found that the patient had stercorolith incarceration in the anastomotic diverticulum, which was tightly wrapped by the diverticulum and could not be removed by irrigation or biopsy forceps. We considered this should be the reason for the patient’s recurrent abdominal pain and the ineffectiveness of conservative treatment. After treated conservatively for about 3 days, the local inflammation was relieved. Then the patient underwent endoscopic mucosal incision and lithotomy, and we closed the diverticulum with titanium clips. During the follow-up 1 year after endoscopic therapy, the abdominal pain of patient was completely relieved. The reexamination of colonoscopy after 1 year also showed that the wound healed well.

This case is important as it is the first reported case of anastomotic diverticulitis caused by stercorolith incarceration. In order to remove the stimulation factor of stercorolith incarceration, we adopted the methods of endoscopic mucosal incision and lithotomy. Compared with conservative treatment, endoscopic therapy can remove the stimulation factors better and avoid the recurrence and progression of the disease. Moreover, this method achieves the maximum in minimally invasive surgery and reduces complications and surgical costs compared with radical surgery. Our report provides novel insights that can serve as a basis for further research and will improve clinicians’ awareness of the clinical and endoscopic manifestations of this special type of anastomotic diverticulitis, resulting in improved diagnosis, treatment, and outcomes.

## Data availability statement

The original contributions presented in this study are included in the article/supplementary material, further inquiries can be directed to the corresponding author.

## Ethics statement

Surgical procedure was approved by the Department of Endoscopy at National Cancer Center/Cancer Hospital, Chinese Academy of Medical Sciences (CICAMS) and the Ethics Committee of National Cancer Center/Cancer Hospital, Chinese Academy of Medical Sciences and Peking Union Medical College (approval number: 18-002/1466). Written informed consent was obtained from the patient for the surgery and for publication of this cohort study and any accompanying images.

## Author contributions

GW: conceptualization, resources, supervision, project administration, and funding acquisition. YiL and ZC: methodology, writing—original draft preparation, and visualization. YiL, LD, and SL: software. ZC, SL, and LD: validation. YiL and YZ: formal analysis. YiL, ZC, and YoL: investigation. YiL and GW: data curation. LD and GW: writing—review and editing. All authors have read and agreed to the published version of the manuscript.
